# Effects of the deletion of the *Escherichia coli *frataxin homologue CyaY on the respiratory NADH:ubiquinone oxidoreductase

**DOI:** 10.1186/1471-2091-8-13

**Published:** 2007-07-24

**Authors:** Thomas Pohl, Julia Walter, Stefan Stolpe, Joel H Defeu Soufo, Peter L Grauman, Thorsten Friedrich

**Affiliations:** 1 Institute of Organic Chemistry and Biochemistry, University of Freiburg, Albertstrasse 21, 79104 Freiburg, Germany; 2 Institute of Microbiology, University of Freiburg, Schänzlestrasse 1, 79104 Freiburg, Germany

## Abstract

**Background:**

Frataxin is discussed as involved in the biogenesis of iron-sulfur clusters. Recently it was discovered that a frataxin homologue is a structural component of the respiratory NADH:ubiquinone oxidoreductase (complex I) in *Thermus thermophilus*. It was not clear whether frataxin is in general a component of complex I from bacteria. The *Escherichia coli *homologue of frataxin is coined CyaY.

**Results:**

We report that complex I is completely assembled to a stable and active enzyme complex equipped with all known iron-sulfur clusters in a *cyaY *mutant of *E. coli*. However, the amount of complex I is reduced by one third compared to the parental strain. Western blot analysis and live cell imaging of CyaY engineered with a GFP demonstrated that CyaY is located in the cytoplasm and not attached to the membrane as to be expected if it were a component of complex I.

**Conclusion:**

CyaY plays a non-essential role in the assembly of complex I in *E. coli*. It is not a structural component but may transiently interact with the complex.

## Background

The NADH:ubiquinone oxidoreductase, also known as respiratory complex I, is the entry point for electrons in the respiratory chains of most bacteria and many eucaryotes. It links the electron transfer from NADH to ubiquinone with the translocation of protons across the membrane. In doing so, complex I establishes a proton motive force required for energy consuming processes [1-5]. One FMN and, depending on the species, eight to nine iron-sulfur (Fe/S) clusters participate in the electron transfer reaction. Generally, the bacterial complex I consists of 14 different subunits called NuoA through NuoN (or Nqo1 through Nqo14; [5-10]). In a few bacteria such as *Escherichia coli *and *Aquifex aeolicus *the genes *nuoC *and *nuoD *are fused resulting in a complex consisting of 13 subunits. Seven (or six, see above) peripheral proteins including those that bear all known redox groups build up the so-called peripheral arm of the complex, which extends into the aqueous medium. The residual seven subunits are hydrophobic proteins and build the membrane arm of the complex. The arrangement of both arms of the complex have been visualized by means of electron microscopy [11,12].

Recently, the structure of the peripheral arm of the complex from *Thermus thermophilus *was resolved at 3.3 Å resolution [13]. This pathbreaking study revealed the unexpected presence of a 15^th ^subunit in the *T. thermophilus *complex [13,14]. The subunit was coined Nqo15 and exhibits structural similarity to the frataxin family. Nqo15 shows a 2.5 Å RMSD to the structure of CyaY, the *E. coli *frataxin homologue [13,15]. Despite the similar three-dimensional fold, the sequence similarity of Nqo15 to members of the frataxin family is very low. Homologues of Nqo15 were only detected in close relatives of *Thermus*, such as *Deinococcus *species [13,14].

Frataxin was first recognized in patients suffering from Friedreich's ataxia [16-18]. Its loss in patients results in a neurodegenerative disease due to an unbalanced iron homeostasis and oxidative damage [16-19]. The exact physiological function of frataxin is still under debate. Frataxin has been shown to specifically albeit weakly bind iron [20] and it was discussed that it is involved in the assembly of Fe/S clusters [21-23]. Recently, it was shown in *E. coli *that CyaY binds to IscS, the cysteine desulfurase of the ISC-system, and delivers iron to the scaffold protein IscU [24]. The deletion of *cyaY *in *E. coli *had no effect on the cellular iron content and its sensitivity to oxidants [25] but it was shown in *Salmonella enterica *that the deletion of *cyaY *in combination with other specific lesions resulted in severe metabolic defects [26].

In this study, we used a *cyaY *deletion mutant to investigate whether or not CyaY is a structural component of the *E. coli *complex I. Our data show that this is not the case but that CyaY is most likely a non-essential component for the assembly of the *E. coli *complex I.

## Results

### Enzymatic activity of complex I

*E. coli *contains two membrane-bound NADH dehydrogenases, the energy-converting complex I and a non-energy-converting, alternative NADH dehydrogenase [9]. While NADH is a substrate for both enzymes the artificial substrate deamino (d)-NADH is only a poor substrate for the alternative dehydrogenase and used to descriminate both enzymes [27,28]. The NADH/ferricyanide oxidoreductase activity of the cytoplasmic membranes was virtually identical in the parental strain and the *cyaY *deletion strain (Table 1). Thus, the total amount of complex I and the alternative NADH dehydrogenase did not differ in the strains.

**Table 1:** Catalytic activities of cytoplasmic membranes from the parental and the *cyaY *deletion strain. The data are the mean of three independent measurements.

Strain	NADH/ferricyanide oxidoreductase activity	NADH oxidase activity	Inhibition by annonin VI	d-NADH oxidase activity	Inhibition by annonin VI
	[μmol· min^-1 ^·mg^-1^]	[μmol· min^-1 ^·mg^-1^]	[%]	[μmol· min^-1· ^mg^-1^]	[%]
BW25113	2.6 ± 0.5	0.29 ± 0.03	52	0.18 ± 0.01	100
BW25113 *cyaY::nptI*	2.7 ± 0.7	0.33 ± 0.06	21	0.13 ± 0.01	100

Strain	Succinate oxidase activity	Inhibition by malonate			
	[μmol· min^-1 ^·mg^-1^]	[%]			

BW25113	0.12 ± 0.01	100			
BW25113 *cyaY::nptI*	0.09 ± 0.01	100			

The physiological NADH oxidase activity of the parental strain was inhibited by 52% by annonin VI, which selectively blocks complex I (Table 1), as observed with other *E. coli *strains [29]. The inhibitor-insensitive activity derived from the alternative NADH dehydrogenase. The d-NADH oxidase activity of this strain, which stems from complex I, was 62% of the activity with NADH as substrate and completely inhibited by annonin VI (Table 1).

Using the *cyaY *deletion strain, an NADH oxidase activity similar to the parental strain was measured, but the inhibition of the activity by annonin VI was approximately 20% (Table 1). This indicated a higher amount of the alternative NADH dehydrogenase in the strain. The d-NADH oxidase activity of the mutant membranes was 39% of the activity with NADH as substrate and 28% lower than the rate measured with the membranes from the parental strain (Table 1). As in the membranes from the parental strain, the d-NADH oxidase activity was fully sensitive to annonin VI, indicating that the d-NADH oxidase activity in this strain derived from complex I. The data demonstrate that a functionally active complex I is present in the cytoplasmic membranes of the *cyaY *deletion mutant, but in a lesser amount.

To investigate whether the *cyaY *deletion had an effect on other Fe/S cluster containing complexes of the *E. coli *respiratory chain, we measured the succinate oxidase activity of the cytoplasmic membranes from both strains. This activity was fully sensitive to malonate (Table 1), a specific inhibitor of the *E. coli *succinate dehydrogenase, the respiratory complex II. The succinate oxidase activity in the mutant membranes was 75% of the activity measured in the membranes of the parental strain (Table 1). Thus, the *cyaY *deletion mutant contains an active succinate dehydrogenase but in lesser amounts. From these measurements it is concluded that the amount of complex I containing nine Fe/S cluster is reduced by one third and the amount of complex II containing three Fe/S clusters by one quarter in the *cyaY *deletion strain.

### Structural integrity of complex I

**Figure 1:**
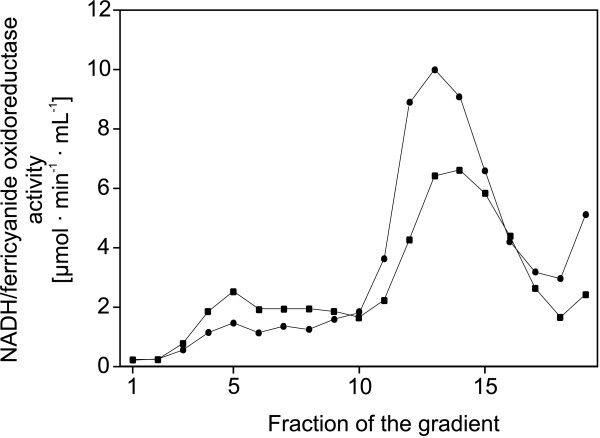
**Sucrose gradient centrifugation of detergent extracts of cytoplasmic membranes**. Cytoplasmic membranes of strains BW25113 (●) and BW25113 *cyaY::nptI *(■) were isolated. Proteins were extracted from cytoplasmic membranes with 3% dodecyl maltoside (w/v) and separated by means of gradients of 5–30% sucrose in 50 mM MES/NaOH, 50 mM NaCl, 5 mM MgCl_2 _and 0.1% dodecyl maltoside, pH 6.0. The gradient with the extract from the parental strain was loaded with 32 mg protein and the gradient with the extract from the mutant membranes with 37 mg protein. The activities shown were calculated to the same amount of 37 mg protein to allow a direct comparison. Fractions of the gradients (numbered 1–20 from top to bottom) were collected and analyzed for NADH/ferricyanide oxidoreductase activity.

The structural integrity of the complex from the parental and the deletion strain was determined by sucrose gradient centrifugation (Fig. 1). Proteins of the cytoplasmic membranes were extracted with 3% (w/v) DDM and centrifuged for 30 min at 150000 × g. The solubilized proteins of the supernatant were separated on a 5–30% (w/v) sucrose gradient by means of ultra-centrifugation for 18 h at 160000 × g. Under these conditions complex I sedimented two thirds of the way through the gradient as indicated by its NADH/ferricyanide oxidoreductase activity ([30,31] Fig. 1). NADH/ferricyanide oxidoreductase activity was detectable in corresponding fractions of the gradient from the parental and the mutant strains indicating that complex I was fully assembled (Fig. 1). The total NADH/ferricyanide oxidoreductase activity of the peak fractions of the mutant strain was two thirds of that of the parental strain. Thus, the amount of complex I in the mutant strain is reduced by approximately one third of that of the parental strain, which is in good agreement with the data obtained from the d-NADH oxidase activity (Table 1).

No enhanced NADH/ferricyanide oxidoreductase activity was detected in fractions of the gradient corresponding to higher molecular masses, revealing that the complex from the *cyaY *deletion strain showed no tendency to aggregate (Fig. 1). No NADH/ferricyanide oxidoreductase activity was detected in fractions 7 to 10 corresponding to the position of a soluble fragment of the complex [30,32], indicating that the complex from the deletion strain did not disintegrate. The NADH/ferricyanide oxidoreductase activity around fraction 5 is due to the alternative NADH dehydrogenase [30]. The activity of this fraction was approximately doubled in the gradient obtained from the *cyaY *deletion strain, demonstrating an enhanced amount of the alternative NADH dehydrogenase in the mutant strain as already indicated by its NADH oxidase activity (Table 1). Thus, a stable complex I was properly assembled in the absence of CyaY.

### Preparation of complex I

**Figure 2:**
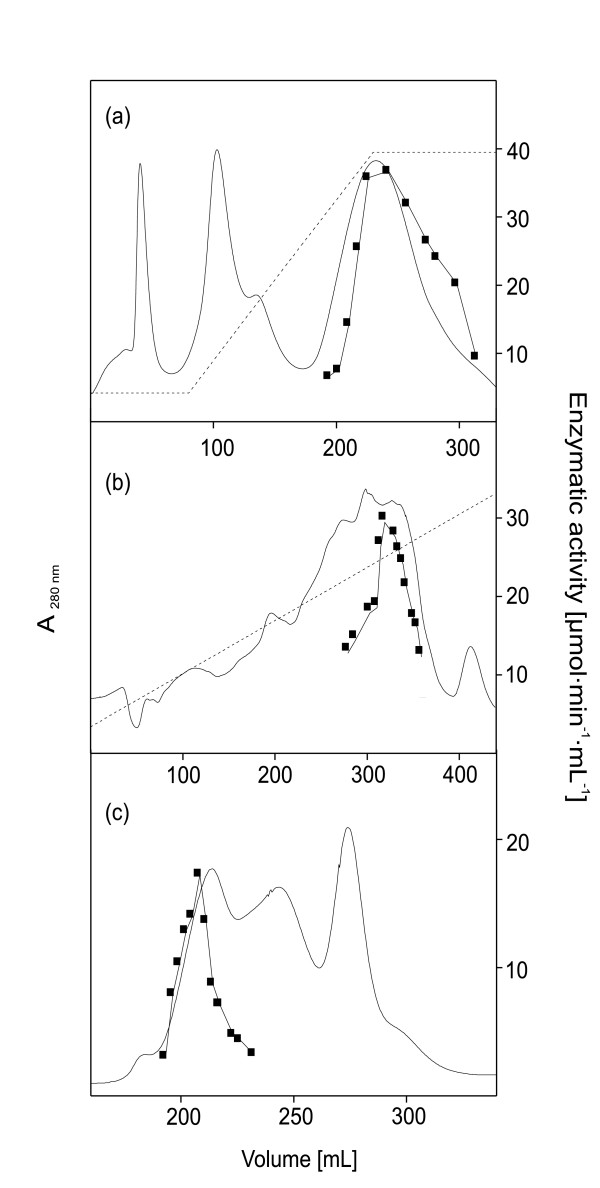
**Complex I preparation**. Isolation of *E. coli *complex I from strain BW25113 *cyaY::nptI*. Chromatography on Fractogel EMD TMAE Hicap M (a); chromatography on Source 15Q (b); chromatography on Sephacryl S-300 HR (c); absorbance at 280 nm (-); NADH/ferricyanide oxidoreductase activity (▪); NaCl gradient (--).

Complex I was isolated from the parental and the mutant strains using a protocol developed in our laboratory (Table 2). Proteins were extracted from the cytoplasmic membranes by DDM and excess detergent was removed by a fast anion-exchange chromatography on EMD-fractogel. Fractions with NADH/ferricyanide oxidoreductase activity were pooled and subjected to a second anion-exchange chromatography on Source 15Q. Peak fractions were pooled and the complex was purified by means of size-exclusion chromatography on Sephacryl S-300 HR (Fig. 2). Complex I eluted from the anion-exchange chromatography on Source 15Q at 220 mM NaCl. The final size-exclusion chromatography on Sephacryl S-300 showed a peak coeluting with the complex I activity at 215 mL (Fig. 2). From both strains 2–3 mg complex I were obtained. Complex I isolated from the parental and the deletion strain was reactivated by addition of phospholipids as described [33]. Both preparations catalyzed electron transfer from NADH to ubiquinone with a rate of 3.0 ± 0.3 μmol NADH min^-1^mg^-1 ^in the presence of 100 μM NADH and 50 μM decyl-ubiquinone. This is similar to the rate obtained with the complex from other *E. coli *strains [29].

**Table 2:** Preparation of complex I. Isolation of *E. coli *complex I from strain BW25113 *cyaY::nptI *starting from 76 g cells (wet weight)*.

Preparation	Volume [mL]	Protein [mg]	NADH/ferricyanide oxidoreductase activity		Yield [%]
				
			total [μmol· min^-1^]	specific [μmol· min^-1 ^·mg^-1^]	
Membranes	280	9100	43530	4.8	100
Extract	64	2010	14680	7.3	34
Fractogel EMD	104	395	3490	9	8
Source 15Q	32	90	1000	11	2
Sephacryl S-300	12	3	137	41	0.3

*Similar values were obtained for the preparation of the complex from the parental strain.

**Figure 3:**
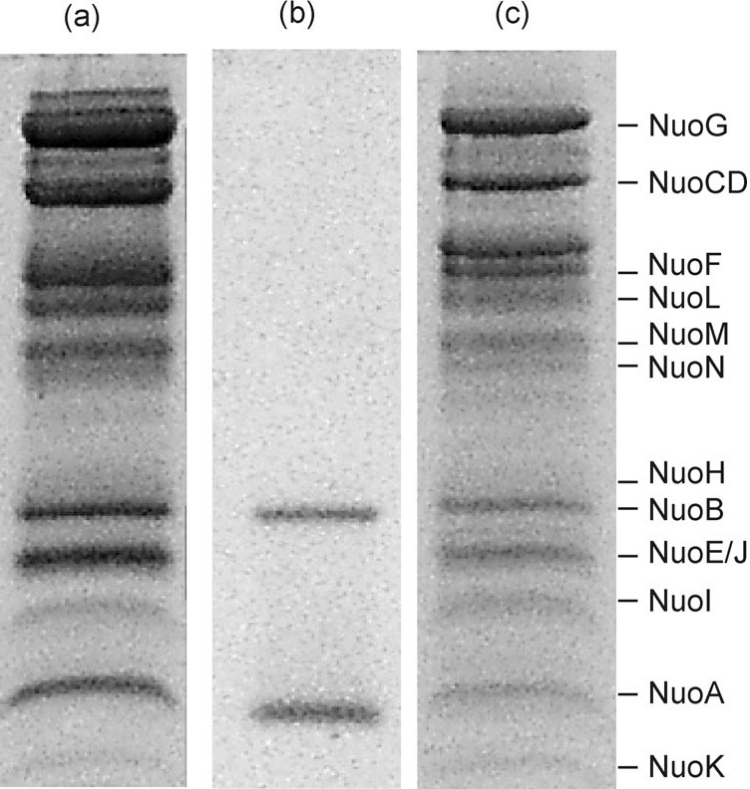
**SDS PAGE analysis**. SDS PAGE of the complex I preparations from the strain BW25113 (a) and BW25113 *cyaY::nptI *(c) and the overproduced and isolated His-tagged CyaY (b). The assignment of the individual bands to the corresponding complex I subunits is given. The band below NuoG represents a degradation product of NuoG. The lane with the preparation from the parental strain was loaded with 100 μg protein, the lane with the preparation from the deletion strain with 80 μg protein. The gel was stained with coomassie R250.

SDS-PAGE of the preparations from the parental strain and the *cyaY *deletion mutant indicated the presence of all complex I subunits (Fig. 3). The subunits NuoE and J were not separated by SDS-PAGE as reported previously [31,34]. The preparation from the deletion strain contained a minor impurity with an apparent molecular mass of about 50 kDa (Fig. 3). An additional subunit with an apparent molecular mass of 12 kDa, the molecular mass of CyaY, was not detectable in the preparation of the complex from the parental strain (Fig. 3).

The CyaY protein decorated with a His-tag was overproduced and purified by affinity chromatography by means of His Spin-Trap on Ni-Sepharose. SDS-PAGE showed the presence of two proteins in the preparation with apparent molecular masses of 12 and 25 kDa, respectively. The molecular mass of CyaY as deduced from its DNA sequence is 12.2 kDa [35]. Thus, the two proteins were attributed to the monomeric and the dimeric form of CyaY, due to a different load with iron [36]. The electrophoretic mobility of the monomeric form of CyaY did not match the mobility of any of the complex I subunits of the preparation from the parental strain (Fig. 3). The CyaY homologue of *T. thermophilus*, Nqo15, was detected in its monomeric form after SDS-PAGE of the peripheral arm of the complex [14]. Thus, none of the obtained data indicated that CyaY could be an integral component of the *E. coli *complex I.

### EPR spectroscopic characterization of complex I

**Figure 4:**
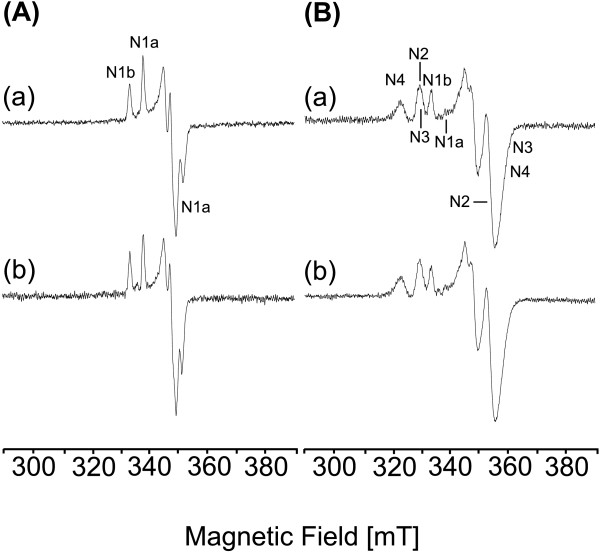
**EPR spectroscopic characterization of complex I**. EPR spectra of complex I isolated from the parental (a) and the deletion strain (b). The spectra shown in (A) were recorded at 40 K and 2 mW microwave power, the spectra shown in (B) were recorded at 13 K and 5 mW microwave power. The signals of the Fe/S clusters N1a, N1b, N2, N3 and N4 are indicated. The signal of cluster N1a is nearly saturated at 13 K and 5 mW. Other EPR conditions were: microwave frequency, 9.44 GHz; modulation amplitude, 0.6 mT; time constant, 0.124 s; scan rate, 17.9 mT/min.

The complex I preparations from the parental and the mutant strain were concentrated to 3 mg/mL and reduced by addition of a 1000-fold molar excess of NADH in the presence of dithionite. EPR spectra recorded at 40 K revealed the contributions of the binuclear Fe/S clusters N1a and N1b, spectra recorded at 13 K contained in addition the signals from the tetranuclear Fe/S clusters N2, N3, and N4 [3,37]. The signals of the Fe/S clusters N1a were detected at g_x,y,z _= 1.92, 1.94, and 2.00 and thoses of N1b at g_//,⊥ _= 2.03 and 1.94 ([38]; Fig. 4). The signal of N1a at g = 2.00 overlaps with a small radical signal due to the reduction by dithionite. Differences concerning the g-values and the amplitudes of the signals were not detectable. The signals of the clusters N2 at g_//,⊥ _= 1.91 and 2.05, N3 at g_x,y,z _= 1.88, 1.92, and 2.04, and N4 at g_x,y,z _= 1.89, 1.93, and 2.09 were present in the spectra of the preparations from the parental strain as well as from the mutant strain ([30,31]; Fig. 4). Thus, differences concerning the Fe/S cluster content and composition were not detectable between the preparations of complex I from the parental and the *cyaY *deletion strain.

### Localization of CyaY by western blot analysis

**Figure 5:**
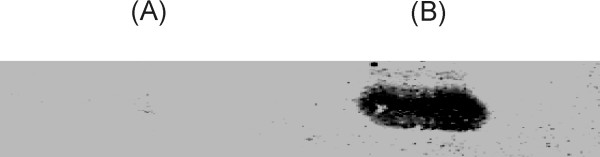
**Localization of CyaY by western blot analysis**. Western blot of the membrane (A) and cytoplasmic (B) fraction of strain BW25113 *cyaY::nptI*/pCA24N*cyaY*. The gel was loaded with 150 μg of membrane proteins and 40 μg of cytoplasmic proteins. Western blotting was performed with an anti-penta-His antibody.

In order to determine the localization of CyaY, the *cyaY *deletion strain was complemented with pCA24N*cyaY *coding for the His-tagged CyaY. After induction with IPTG, cells were grown to the late exponential phase, collected by centrifugation and washed twice with 50 mM MES/NaOH pH 6.0. The cells were broken by a single pass through a french pressure cell, the cell debris was removed by centrifugation and the cytoplasmic and the membrane fraction separated by ultra-centrifugation at 250000 × g for 1 h. The membranes were resuspended and washed three times in 50 mM MES/NaOH, 50 mM NaCl, pH 6.0. Proteins of the cytoplasmic and the membrane fraction were separated by SDS-PAGE and blotted onto a nitrocellulose membrane. The His-tagged CyaY was detected by an antibody directed against the His-tag (Fig. 5). A clear signal corresponding to a protein of a molecular mass of 12 kDa was detected in the cytoplasmic fraction but not in the membrane fraction (Fig. 5). Thus, at least the vast majority of CyaY is located in the cytoplasmic fraction and not associated with the membrane.

The overproduced CyaY modified with a His-tag was capable of binding 6.7 ± 0.5 mol Fe^3+^/mol CyaY under aerobic conditions. It has been shown that recombinant CyaY can bind eight Fe^3+ ^[20]. Thus, failure to detect CyaY within the membrane fraction is not due to a distorted Fe-binding of the recombinant protein.

### Life cell imaging of CyaY

**Figure 6:**
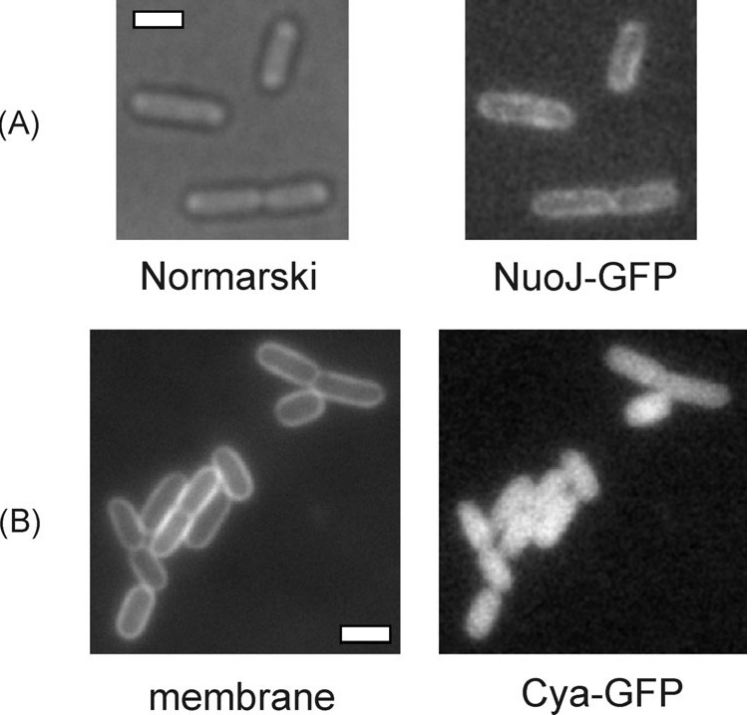
**Life cell imaging**. Fluorescence microscopy of growing *E. coli *cells. A) Nomarski DIC (bright field) image and GFP channel of cells expressing NuoJ-GFP, B) FM4-64 stained membranes or GFP fluorescence of cells expressing CyaY-GFP. White bars 2 μm.

If CyaY was a structural component of the *E. coli *complex I it should be preferentially located close to the membrane. In another approach to determine the cellular location of CyaY, a GFP-fusion of the protein was overproduced in the *cyaY *deletion strain. As a control, a NuoJ-GFP fusion was expressed from a plasmid, which resulted in a clear fluorescent stain of the *E. coli *inner membrane, but not of the cytosol (Fig. 6A). The *cyaY-gfp *construct was expressed under the control of the *araBAD*-promotor of pBAD. After induction with arabinose the cells showed green fluorescence (Fig. 6B), which was greatly diminished by repressing the expression with glucose (data not shown). The GFP fluorescence was regularly distributed throughout the cytoplasm. No clustering of the fluorescence close to the membrane comparable to that of NuoJ-GFP was observed. The membrane was stained with the vital dye FM4-64 (Fig. 6). Thus, CyaY is *in vivo *not attached to the cytoplasmic membrane and therefore not a structural component of complex I.

## Discussion

The previously unrecognized protein Nqo15 was found to be a structural component of the *T. thermophilus *complex I [13,14]. This soluble protein was co-purified with the preparation of the peripheral arm of the respiratory complex I from the membrane fraction of the disrupted cells. It co-eluted with the peripheral arm from four different chromatographic steps, and SDS-PAGE of the preparation revealed that it was present in stoichiometric amounts [14]. Finally, the crystal structure of the peripheral arm unambiguously showed that this subunit is an integral part of the *T. thermophilus *complex I [13].

The genes comprising the structural subunits of the bacterial complex I are for the most part organized in one operon or in one or more gene clusters [6,7]. The complex I gene clusters in *Rhodobacter capsulatus *[39] and *Paracoccus denitrificans *[9] contain additional ORFs that are not related to each other. These ORFs do not encode known complex I subunits and it has been shown that a disruption of the ORFs has no influence on the assembly and the enzymatic activity of complex I [5,39]. The gene coding the CyaY homologue in *T. thermophilus *is neither located within nor in close proximity to the complex I gene cluster. Thus, the finding of this protein as an integral complex I component was completely unexpected [14].

The assignment of Nqo15 to the family of frataxin single domain proteins (cd00503) was not obvious from its primary structure. Solely the crystal structure revealed that Nqo15 exhibits the typical fold of a member of the frataxin family [13]. Electron density for bound iron was not detected in the structure, implying that the metal might have been lost during purification. Here, we have shown that the frataxin homologue of *E. coli *CyaY is not a structural component of complex I. The preparation of complex I from the wild type did not contain a subunit of an apparent molecular mass corresponding to the molecular mass of CyaY (Fig. 3). The complex I was fully assembled in the membrane of the mutant and contains all subunits and known cofactors (Table 1; Figs. 1, 3, and 4). Its physiological NADH:decyl-ubiquinone oxidoreductase activity was indistinguishable from the activity of the complex I preparation from wild type. We were able to verify the analyses *in vivo*, because GFP-labelled CyaY did not associate with the membrane like NuoJ, but showed a cytosolic localization. Thus, it is reasonable to assume that CyaY is not a structural component of the *E. coli *complex I.

An important finding in this study is that the amount of complex I in the mutant was reduced by one third as derived from the inhibitor-sensitive portion of the NADH oxidase activity and the d-NADH oxidase activity of the membranes (Table 1) as well as from the sucrose gradient centrifugation of detergent extracts (Fig. 1). The overall NADH dehydrogenase activity of the membranes did not change as indicated by the unaltered NADH/ferricyanide oxidoreductase activity (Table 1). These data demonstrate that the amount of the alternative NADH dehydrogenase is increased approximately two-fold. Thus, the ratio of complex I to the alternative enzyme changed due to the deletion of *cyaY*. This might be due to a change of the metabolic and/or redox state of the mutant cells. However, it is well known that mutations in the complex I genes leading to a decreased amount of the complex or the assembly of an inactive complex result in a two-fold enhanced production of the alternative enzyme [10,27,29,30]. Therefore, it seems rather unlikely that the deletion of *cyaY *changed the properties of the cell leading to a subsequent change of the ratio of complex I to the alternative NADH dehydrogenase.

The deletion of *cyaY *led to a decrease of the complex I and complex II content of the cytoplasmic membrane by approximately one third and one quarter, respectively (Table 1). This implies a transient interaction between CyaY and the complexes of the *E. coli *respiratory chain. It is possible that CyaY is involved in either biogenesis or repair of the Fe/S clusters of the complexes containing Fe/S clusters. The stoichiometry of the individual Fe/S clusters in complex I were not distinguishable in the preparation from wild type and the *cyaY *deletion strain (Fig. 4). If CyaY played a role in the repair of Fe/S clusters this would imply that complex I with damaged Fe/S clusters has to be degraded in the mutant membranes.

It was proposed that CyaY is not involved in the biogenesis of Fe/S clusters but may serve as an iron chaperone binding cellular iron in order to reduce oxidative damage under oxidative stress [40]. According to this CyaY binds redox active iron and prevents the formation of hydrogen peroxide [40]. A deletion of CyaY leads to an increased cellular amount of reactive oxygen species which subsequently destroys Fe/S clusters in iron-sulfur proteins [40]. If the oxidative damage of the Fe/S clusters due to the deletion of CyaY leads to the reduced content of complex I all clusters of the complex would be concerned to the same extent, as the stoichiometry of the individual Fe/S clusters is virtually identical in the preparation from wild type and the *cyaY *deletion strain (Fig. 4). This is rather unlikely because the accessibility of the Fe/S clusters of complex I and the polarity of their environment differ significantly [13]. If CyaY played a role in preventing a damage of the Fe/S clusters this would again imply that complex I with damaged Fe/S clusters has to be degraded in the mutant membranes. However, it has been demonstrated that complex I with a reduced content of a single Fe/S cluster is stable in the membrane [29,38].

Thus, it is more likely that CyaY is one component of the machinery of Fe/S cluster biogenesis as proposed [24,41,42]. Formation of Fe/S clusters require complex biosynthesis systems which share the involvement of cysteine desulfurases and Fe/S cluster scaffold proteins [42,43]. The desulfurase catalyzes the reductive conversion of cysteine to alanine and sulfide. Together with iron, the sulfide is assembled to a binuclear Fe/S cluster on the scaffold protein. The dimer of the scaffold protein is able to build a tetranuclear Fe/S cluster out of the two binuclear clusters [44]. Due to the low solubility of iron and its toxicity in the cell, the presence of an iron donor protein is most likely. The role of the protein would be to bind and hence solubilize the iron, and to transport and deliver it to the desulfurase/scaffold proteins. In this respect it should not be essential for the biosynthetic reaction. There is evidence that frataxin is the protein sought after. It was shown that frataxins are able to bind iron and exist in oligomeric forms, which seem to be the active species [20,24,44-47] and that they interact with the scaffold protein and the desulfurase [23,24]. In agreement with the proposed function of the iron donor protein, it was shown that the deletion of the corresponding genes in yeast and in *E. coli *is not lethal [21,25]. In accordance with these data, the deletion of *cyaY *leads to a reduced content of complex I and II in *E. coli*, most likely due to a reduced biogenesis of their Fe/S clusters, but it did not significantly change the growth rate of the mutant strain.

## Conclusion

The *E. coli *frataxin homologue CyaY is located in the cytoplasm and is therefore not a structural component of complex I. A *cyaY *deletion mutant showed a reduced complex I and complex II production which may be a result of the disturbance of the Fe/S cluster assembly machinery. Further studies are necessary to determine if a transient interaction between the complexes of the respiratory chain and CyaY takes place during the biogenesis of the Fe/S clusters.

## Methods

### Strains, plasmids and gene expression

*E. coli *K-12 strains AG1, BW25113 and BW25113 *cyaY::nptI *were kindly provided by the Keio collection of the Nara Institute of Science and Technology (National BioResource Project (NIG, Japan): *E. coli*) [48]. The plasmids pCA24N*cyaY *and pCA24N*cyaY-gfp *were obtained from the ASKA library [49]. They contain the sequence coding for CyaY with an N-terminal 6× histidine (His)-tag (pCA24N*cyaY*) and an additional C-terminal GFP-fusion (pCA24N*cyaY-gfp*). The plasmid pGFPe *nuoJ *contains *nuoJ *engineered with a C-terminal GFP-fusion and was kindly provided by Drs. Gunnar von Heijne and Daniel Daley [50]. The expression host BL21(DE3) for pGFPe *nuoJ *was purchased from Novagen. Strains were grown in LB medium at 37°C until early stationary phase. Cells used for fluorescence microscopy were grown in M9 minimal medium with 30 mM mannitol as carbon source at 25°C. Chloramphenicol (100 μg/mL) and kanamycin (50 μg/ml) were supplemented when necessary. Expression of pBAD*cyaY-gfp *(see below) was induced by adding 0.2% (w/v) L-arabinose to the media and repressed in the presence of 0.2% (w/v) D-glucose. Expression of pCA24N*cyaY *and pGFPe *nuoJ *was induced by an addition of 1 mM isopropyl-*β*-D-thiogalactopyranoside.

### Cloning of pBAD*cyaY-gfp*

The *cyaY-gfp *fusion was PCR-amplified from pCA24N*cyaY-gfp *using Phusion DNA Polymerase (Finnzymes) and primers *XbaI-cyaY *(5'AGTTCTAGAAGG AGGAATTCACCATGAACACAGTGAATTTCATCGCCTG) and *gfp-HindIII *(5'-AGTAAGCTTGCAGGTCGACCCTTAGCG) and cloned in pBAD33 [51] using the same primers. The forward primer contains a synthetic ribosomal binding site AGGAGG 8 nt upstream of the initiation codon. The PCR product was cut with *XbaI *and *HindIII *and ligated to *XbaI-HindIII *sites of pBAD33 downstream of the *araBAD *promotor.

### Protein purification

Complex I was isolated similarly to the procedure described [33]. All steps were carried out at 4°C. 76 g cells were resuspended in a 5-fold volume of 50 mM MES/NaOH, 0.1 mM phenylmethanesulfonyl fluoride, pH 6.0, with 10 μg/mL DNAseI and 50 μg/mL lysozyme and disrupted by a single pass through a French Pressure cell (SLM Aminco) at 110 MPa. Cell debris was removed by centrifugation at 36000 × g for 20 min and cytoplasmic membranes were obtained by centrifugation at 250000 × g for 1 h. The membranes were resuspended in 50 mM MES/NaOH, 50 mM NaCl, pH 6.0 at a protein concentration of 80 mg/mL. *n*-Dodecyl-*β*-D-maltopyranoside (DDM, AppliChem) was added to a final concentration of 3% and the solution was gently homogenized and centrifuged for 20 min at 250000 × g. The supernatant was applied to a 120 mL Fractogel EMD TMAE Hicap M (Merck) column equilibrated in 50 mM MES/NaOH, 50 mM NaCl and 0.1% DDM, pH 6.0. The column was eluted with a 150 mL linear gradient of 150–350 mM NaCl in 50 mM MES/NaOH, 0.1% DDM, pH 6.0 at a flow rate of 15 mL/min. Fractions containing NADH/ferricyanide oxidoreductase activity were combined, concentrated by precipitation with 9% (w/v; final concentration) poly(ethylene glycol) 4000 and dissolved in 5 mL 50 mM MES/NaOH, 50 mM NaCl and 0.1% DDM, pH 6.0. The proteins were loaded onto a 80 mL Source 15Q (GE Healthcare) column equilibrated in 50 mM MES/NaOH, 50 mM NaCl and 0.1% DDM, pH 6.0. The column was eluted with a 500 mL linear gradient of 125–275 mM NaCl in 50 mM MES/NaOH, 0.1% DDM, pH 6.0 at a flow rate of 5 mL/min. Fractions containing NADH/ferricyanide oxidoreductase activity were pooled and concentrated with a 100 kDa MWCO Amicon Ultra-15 centrifugal filter (Millipore). The concentrated protein solution was subjected to size-exclusion chromatography on a 450 mL Sephacryl S-300 HR (GE Healthcare) column in 50 mM MES/NaOH, 50 mM NaCl, and 0.1% DDM, pH 6.0, at a flow rate of 20 mL/h. Peak fractions of NADH/ferricyanide oxidoreductase activity were combined and stored at -80°C.

His-tagged CyaY was isolated from strain AG1/pCA24N*cyaY*. For SDS-PAGE analysis 0.24 g wet cells were treated with Bugbuster protein extraction reagent (Novagen) according to manufacturer's recommendations. Cell debris and lipids were removed by centrifugation at 150000 × g for 15 min at 4°C and the supernatant was adjusted to 20 mM imidazole and loaded onto a 100 μL His Spin-Trap column (GE Healthcare). After washing with 600 μL 20 mM imidazole in 20 mM Na_3_PO_4_/HCl pH 7.4 and 600 μL 500 mM imidazole in 20 mM Na_3_PO_4_/HCl pH 7.4 the protein was eluted with 600 μL 1 M imidazole in 20 mM Na_3_PO_4_/HCl pH 7.4.

For determination of iron binding capacity His-tagged CyaY was isolated from 2 g wet cells. All steps were carried out at 4°C. The cells were resuspended in a 10-fold volume of 20 mM Na_3_PO_4_/HCl, 20 mM imidazole, 500 mM NaCl 0.1 mM phenylmethanesulfonyl fluoride, pH 7.4 (binding buffer), with 10 μg/mL DNAseI and 50 μg/mL lysozyme and disrupted by a single pass through a French Pressure cell (SLM Aminco) at 110 MPa. Cell debris and membranes were removed by centrifugation at 250000 × g for 1 h. The supernatant was loaded onto a 15 mL ProBond Ni^2+^-IDA column equilibrated in binding buffer. The column was washed with 80 mL of binding buffer and proteins were eluted with a 60 mL linear gradient of 20–1000 mM imidazole in binding buffer. Fractions were analyzed by SDS-PAGE and those containing His-tagged CyaY were combined and concentrated by a 3 kDa MWCO Vivaspin 20 centrifugal filter (Vivascience).

### EPR spectroscopy

EPR spectroscopy was performed with a Bruker EMX 1/6 spectrometer operating at X-band (9.2 GHz) according to [30]. The magnetic field was calibrated using a strong or a weak pitch standard. The isolated complex I (3 mg/mL) was reduced with a few grains of dithionite in the presence of a 1000-fold molar excess of NADH.

### Enzyme activity

Complex I activity in the cytoplasmic membranes was measured either as NADH/ferricyanide oxidoreductase activity or as d-NADH oxidase activity as described [27,29]. The d-NADH oxidase activity was inhibited by addition of 20 μM annonin VI, a specific complex I inhibitor [27]. The NADH:decyl-ubiquinone oxidoreductase activity of isolated complex I was determined as described [31]. The succinate oxidase activity of cytoplasmic membranes was measured with a clark oxygen electrode. To remove tightly bound oxaloacetate at the active site and to express the full catalytic activity of succinate dehydrogenase, cytoplasmic membranes corresponding to 600 μg protein were incubated for 10 min in 2 mL 30 mM Na_2_HPO_4_/HCl pH 7.4 at 30°C [52]. The reaction was initiated by the addition of 20 mM succinate and inhibited in the presence of 40 mM malonate.

### Iron binding to modified CyaY

The binding of iron to the overproduced and His-tagged CyaY was determined according to [24]. Purified CyaY was incubated with a 15-fold molar excess of Fe^3+ ^in form of ferric ammonium citrate under aerobic conditions at 4°C for 1 hour. The mixtures were centrifuged at 16000 × g and desalted on a 20 mL Sephadex G25 superfine column (Amersham Pharmacia) equilibrated in 50 mM Tris/HCl, 50 mM NaCl, pH 7.0. The iron content of the preparation before and after loading with Fe^3+ ^was determined according to [53].

### Fluorescence microscopy

Cells at mid-exponential phase were placed on a microscope slide covered with a pad of 1% (w/v) agarose in M9-mannitol minimal media. A cover slip was placed on the cells and images were acquired with a Axio Imager A.1 fluorescence microscope (Zeiss) at 1000× magnification. Pictures were acquired with a digital CCD camera and processed with Metamorph 4.6 (Universal Imagin Corp., USA). The cytoplasmic membranes were stained with 1 nM N-(3-triethylammoniumpropyl)-4-(p-diethylaminophenylhexatrienyl)pyridinium dibromide (FM4-64).

### Other Analytical Procedures

Protein concentration was measured either by the biuret or the Bradford method [54] using BSA as standard. SDS-PAGE was performed according to the protocol of Schägger and von Jagow [55], using a 10% T, 3% C separating gel. The concentration of the isolated proteins was determined by the absorbance at 280 nm using an extinction coefficient of 764 mM^-1^cm^-1 ^for complex I and 30 mM^-1^cm^-1 ^for CyaY derived from their DNA sequence. Sucrose-gradient centrifugation in the presence of 0.1% DDM was performed as described [30]. Proteins separated by SDS-PAGE were electroblotted onto 0.45 μm pore size nitrocellulose membrane (Schleicher und Schüll) for western blot analysis [56]. An Anti-Penta-His antibody (Qiagen) was used at a 1:400 dilution for detection. The membrane was incubated for 1 h at 25°C with the primary antibody.

## Abbreviations

The abbreviations used are: Complex I, proton-pumping NADH:ubiquinone oxidoreductase; d-NADH, deamino-NADH; DDM, n-Dodecyl-*β*-D-maltopyranoside; decyl-ubiquinone, 2,3-dimethoxy-5-methyl-6-decyl-benzoquinone; EPR, electron paramagnetic resonance; FMN, flavin mononucleotide; Fe/S, iron-sulfur; MES, 2-(N-morpholino)-ethanesulfonic acid; Tris, tris-(hydroxymethyl)aminomethane

## Authors' contributions

TP and TF conceived and designed the study and drafted the manuscript. JW performed the activity assays, western blot analysis, cloning and iron binding assay. SS purified complex I. TF carried out the EPR spectroscopy measurements. JHDS performed the fluorescence microscopy. PLG edited the fluorescence microscopy pictures and helped draft the manuscript. All authors read and approved the final manuscript.

## References

[B1] Walker JE (1992). The NADH:ubiquinone oxidoreductase (complex I) of respiratory chains. Q Rev Biophys.

[B2] Weiss H, Friedrich T, Hofhaus G, Preis D (1991). The respiratory-chain NADH dehydrogenase (complex I) of mitochondria. Eur J Biochem.

[B3] Ohnishi T (1998). Iron-sulfur clusters/semiquinones in complex I. Biochim Biophys Acta.

[B4] Brandt U, Kerscher S, Dröse S, Zwicker K, Zickermann V (2003). Proton pumping by NADH:ubiquinone oxidoreductase. A redox driven conformational change mechanism?. FEBS Lett.

[B5] Yagi T, Matsuno-Yagi A (2003). The proton-translocating NADH-quinone oxidoreductase in the respiratory chain: the secret unlocked. Biochemistry.

[B6] Friedrich T, Steinmüller K, Weiss H (1995). The proton-pumping respiratory complex I of bacteria and mitochondria and its homologue in chloroplasts. FEBS Lett.

[B7] Friedrich T, Scheide D (2000). The respiratory complex I of bacteria, archaea and eukarya and its module common with membrane-bound multisubunit hydrogenases. FEBS Lett.

[B8] Friedrich T (2001). Complex I: a chimaera of a redox and conformation-driven proton pump?. J Bioenerg Biomembr.

[B9] Yagi T, Yano T, Di Bernado S, Matsuno-Yagi A (1998). Procaryotic complex I (NDH-1), an overview. Biochim Biophys Acta.

[B10] Friedrich T (1998). The NADH:ubiquinone oxidoreductase (complex I) from *Escherichia coli*. Biochim Biophys Acta.

[B11] Grigorieff N (1999). Structure of the respiratory NADH:ubiquinone oxidoreductase (complex I). Curr Opin Struct Biol.

[B12] Friedrich T, Böttcher B (2004). The gross structure of the respiratory complex I: a Lego System. Biochim Biophys Acta.

[B13] Sazanov LA, Hinchliffe P (2006). Structure of the hydrophilic domain of respiratory complex I from *Thermus thermophilus*. Science.

[B14] Hinchliffe P, Carroll J, Sazanov LA (2006). Identification of a novel subunit of respiratory complex I from *Thermus thermophilus*. Biochemistry.

[B15] Cho SJ, Lee MG, Yang JK, Lee JY, Song HK, Suh SW (2000). Crystal structure of *Escherichia coli *CyaY protein reveals a previously unidentified fold for the evolutionarily conserved frataxin family. Proc Natl Acad Sci USA.

[B16] Chamberlain S, Shaw J, Rowland A, Wallis J, South S, Nakamura Y, von GA, Farrall M, Williamson R (1988). Mapping of mutation causing Friedreich's ataxia to human chromosome 9. Nature.

[B17] Campuzano V, Montermini L, Molto MD, Pianese L, Cossee M, Cavalcanti F, Monros E, Rodius F, Duclos F, Monticelli A, Zara F, Canizares J, Koutnikova H, Bidichandani SI, Gellera C, Brice A, Trouillas P, De Michele G, Filla A, De Frutos R, Palau F, Patel PI, Di Donato S, Mandel JL, Cocozza S, Koenig M, Pandolfo M (1996). Friedreich's ataxia: autosomal recessive disease caused by an intronic GAA triplet repeat expansion. Science.

[B18] Campuzano V, Montermini L, Lutz Y, Cova L, Hindelang C, Jiralerspong S, Trottier Y, Kish SJ, Faucheux B, Trouillas P, Authier FJ, Durr A, Mandel JL, Vescovi A, Pandolfo M, Koenig M (1997). Frataxin is reduced in Friedreich ataxia patients and is associated with mitochondrial membranes. Hum Mol Genet.

[B19] Pandolfo M (2002). The molecular basis of Friedreich ataxia. Adv Exp Med Biol.

[B20] Bou-Abdallah F, Adinolfi S, Pastore A, Laue TM, Dennis CN (2004). Iron binding and oxidation kinetics in frataxin CyaY of *Escherichia coli*. J Mol Biol.

[B21] Babcock M, de Silva D, Oaks R, Davis-Kaplan S, Jiralerspong S, Montermini L, Pandolfo M, Kaplan J (1997). Regulation of mitochondrial iron accumulation by Yfh1p, a putative homolog of frataxin. Science.

[B22] Duby G, Foury F, Ramazzotti A, Herrmann J, Lutz T (2002). A non-essential function for yeast frataxin in iron-sulfur cluster assembly. Hum Mol Genet.

[B23] Gerber J, Mühlenhoff U, Lill R (2003). An interaction between frataxin and Isu1/Nfs1 that is crucial for Fe/S cluster synthesis on Isu1. EMBO Rep.

[B24] Layer G, Ollagnier-de Choudens S, Sanakis Y, Fontecave M (2006). Iron-sulfur cluster biosynthesis: characterization of *Escherichia coli* CYaY as an iron donor for the assembly of [2Fe-2S] clusters in the scaffold IscU. J Biol Chem.

[B25] Li DS, Ohshima K, Jiralerspong S, Bojanowski MW, Pandolfo M (1999). Knock-out of the cyaY gene in *Escherichia coli *does not affect cellular iron content and sensitivity to oxidants. FEBS Lett.

[B26] Vivas E, Skovran E, Downs DM (2006). Salmonella enterica strains lacking the frataxin homolog CyaY show defects in Fe-S cluster metabolism in vivo. J Bacteriol.

[B27] Friedrich T, van Heek P, Leif H, Ohnishi T, Forche E, Kunze B, Jansen R, Trowitzsch-Kienast W, Höfle G, Reichenbach H, Weiss H (1994). Two binding sites of inhibitors in NADH: ubiquinone oxidoreductase (complex I). Relationship of one site with the ubiquinone-binding site of bacterial glucose:ubiquinone oxidoreductase. Eur J Biochem.

[B28] Matsushita K, Ohnishi T, Kaback HR (1987). NADH-ubiquinone oxidoreductases of the *Escherichia coli *aerobic respiratory chain. Biochemistry.

[B29] Flemming D, Hellwig P, Friedrich T (2003). Involvement of tyrosines 114 and 139 of subunit NuoB in the proton pathway around cluster N2 in *Escherichia coli* NADH:ubiquinone oxidoreductase. J Biol Chem.

[B30] Leif H, Sled VD, Ohnishi T, Weiss H, Friedrich T (1995). Isolation and characterization of the proton-translocating NADH: ubiquinone oxidoreductase from *Escherichia coli*. Eur J Biochem.

[B31] Spehr V, Schlitt A, Scheide D, Guénebaut V, Friedrich T (1999). Overexpression of the *Escherichia coli **nuo*-operon and isolation of the overproduced NADH:ubiquinone oxidoreductase (complex I). Biochemistry.

[B32] Braun M, Bungert S, Friedrich T (1998). Characterization of the overproduced NADH dehydrogenase fragment of the NADH:ubiquinone oxidoreductase (complex I) from *Escherichia coli*. Biochemistry.

[B33] Stolpe S, Friedrich T (2004). The *Escherichia coli* NADH:ubiquinone oxidoreductase (complex I) is a primary proton pump but may be capable of secondary sodium antiport. J Biol Chem.

[B34] Sazanov LA, Carroll J, Holt P, Toime L, Fearnley IM (2003). A role for native lipids in the stabilization and two-dimensional crystallization of the *Escherichia coli* NADH-ubiquinone oxidoreductase (complex I). J Biol Chem.

[B35] Huynen MA, Snel B, Bork P, Gibson TJ (2001). The phylogenetic distribution of frataxin indicates a role in iron-sulfur cluster protein assembly. Hum Mol Genet.

[B36] Adinolfi S, Trifuoggi M, Politou AS, Martin S, Pastore A (2002). A structural approach to understanding the iron-binding properties of phylogenetically different frataxins. Hum Mol Genet.

[B37] Ohnishi T, Sled VD, Yano T, Yagi T, Burbaev DS, Vinogradov AD (1998). Structure-function studies of iron-sulfur clusters and semiquinones in the NADH-Q oxidoreductase segment of the respiratory chain. Biochim Biophys Acta.

[B38] Uhlmann M, Friedrich T (2005). EPR signals assigned to Fe/S cluster N1c of the *Escherichia coli* NADH:ubiquinone oxidoreductase (complex I) derive from cluster N1a. Biochemistry.

[B39] Dupuis A, Chevallet M, Darrouzet E, Duborjal H, Lunardi J, Issartel JP (1998). The complex I from *Rhodobacter capsulatus*. Biochim Biophys Acta.

[B40] Ding H, Yang J, Coleman LC, Yeung S (2007). Distinct Iron Binding Property of Two Putative Iron Donors for the Iron-Sulfur Cluster Assembly. J Biol Chem.

[B41] Yoon T, Cowan JA (2003). Iron-sulfur cluster biosynthesis. Characterization of frataxin as an iron donor for assembly of [2Fe-2S] clusters in ISU-type proteins. J Am Chem Soc.

[B42] Lill R, Mühlenhoff U (2005). Iron-sulfur-protein biogenesis in eukaryotes. Trends Biochem Sci.

[B43] Johnson DC, Dean DR, Smith AD, Johnson MK (2005). Structure, function, and formation of biological iron-sulfur clusters. Annu Rev Biochem.

[B44] Agar JN, Krebs C, Frazzon J, Huynh BH, Dean DR, Johnson MK (2000). IscU as a scaffold for iron-sulfur cluster biosynthesis: sequential assembly of [2Fe-2S] and [4Fe-4S] clusters in IscU. Biochemistry.

[B45] O'Neill HA, Gakh O, Isaya G (2005). Supramolecular assemblies of human frataxin are formed via subunit-subunit interactions mediated by a non-conserved amino-terminal region. J Mol Biol.

[B46] Aloria K, Schilke B, Andrew A, Craig EA (2004). Iron-induced oligomerization of yeast frataxin homologue Yfh1 is dispensable in vivo. EMBO Rep.

[B47] Cook JD, Bencze KZ, Jankovic AD, Crater AK, Busch CN, Bradley PB, Stemmler AJ, Spaller MR, Stemmler TL (2006). Monomeric yeast frataxin is an iron-binding protein. Biochemistry.

[B48] Baba T, Ara T, Hasegawa M, Takai Y, Okumura Y, Baba M, Datsenko KA, Tomita M, Wanner BL, Mori H (2006). Construction of *Escherichia coli* K-12 in-frame, single-gene knockout mutants: the Keio collection. Mol Syst Biol.

[B49] Kitagawa M, Ara T, Arifuzzaman M, Ioka-Nakamichi T, Inamoto E, Toyonaga H, Mori H (2005). Complete set of ORF clones of *Escherichia coli* ASKA library (A Complete Set of *E. coli* K-12 ORF Archive): Unique Resources for Biological Research. DNA Res.

[B50] Rapp M, Drew D, Daley DO, Nilsson J, Carvalho T, Melen K, De Gier JW, Von Heijne G (2004). Experimentally based topology models for *E. coli* inner membrane proteins. Protein Sci.

[B51] Guzman LM, Belin D, Carson MJ, Beckwith J (1995). Tight regulation, modulation, and high-level expression by vectors containing the arabinose PBAD promoter. J Bacteriol.

[B52] Maklashina E, Cecchini G (1999). Comparison of catalytic activity and inhibitors of quinone reactions of succinate dehydrogenase (succinate-ubiquinone oxidoreductase) and fumarate reductase (menaquinol-fumarate oxidoreductase) from *Escherichia coli*. Arch Biochem Biophys.

[B53] Percival MD (1991). Human 5-lipoxygenase contains an essential iron. J Biol Chem.

[B54] Bradford MM (1976). A rapid and sensitive method for the quantitation of microgram quantities of protein utilizing the principle of protein-dye binding. Anal Biochem.

[B55] Schägger H, von Jagow G (1987). Tricine-sodium dodecyl sulfate-polyacrylamide gel electrophoresis for the separation of proteins in the range from 1 to 100 kDa. Anal Biochem.

[B56] Towbin H, Staehelin T, Gordon J (1979). Electrophoretic transfer of proteins from polyacrylamide gels to nitrocellulose sheets: procedure and some applications. Proc Natl Acad Sci USA.

